# Staged open ascending aortic replacement and endovascular total arch repair using a three-inner-branch physician-modified endograft for chronic postdissection aneurysms

**DOI:** 10.1016/j.jvscit.2026.102527

**Published:** 2026-09-20

**Authors:** William J. Yoon, Alexander P. Nissen

**Affiliations:** a Department of Vascular Surgery, MedStar Washington Hospital Center–Georgetown University Hospital, Washington, DC; b Department of Surgery, Georgetown University School of Medicine, Washington, DC; c The Section of Vascular Surgery, Department of Surgical Sciences, Uppsala University, Uppsala, Sweden; d Department of Cardiac Surgery, MedStar Washington Hospital Center, Washington, DC

**Keywords:** Chronic postdissection, Aneurysm, Branched endograft, Endovascular procedure

## Abstract

This report describes a single-admission, two-stage hybrid approach for chronic postdissection aneurysmal degeneration of the ascending aorta and aortic arch. Stage 1 consists of open ascending aortic replacement without deep hypothermic circulatory arrest. Stage 2 uses a three-inner-branch physician-modified endograft with novel features for total arch repair, relying on the surgical graft from stage 1 as the proximal landing zone. To minimize cardiac pulsatility-induced motion, atriocaval occlusion was used, alongside a repurposed thoracic branch endoprosthesis side branch for secure innominate artery sealing. This less invasive strategy simplifies arch reconstruction and broadens treatment options for high-risk patients with postdissection multisegment aneurysms.

Although open surgery remains the gold standard for complex chronic postdissection aneurysmal disease involving the ascending aorta and aortic arch,^1^^,^^2^ management requires individualized strategies based on patient anatomy and physiologic reserve. When deep hypothermic circulatory arrest and supra-aortic debranching pose prohibitive surgical risks, total endovascular repair using branched or fenestrated devices provides a viable alternative. Given the limited availability of triple-vessel devices dedicated to total aortic arch repair, physician-modified endografts (PMEGs) remain indispensable.^3^ We present a single-admission, anatomy-driven, two-stage hybrid procedure for these cases: open ascending aorta repair without hypothermic circulatory arrest, followed by endovascular total aortic arch reconstruction using a three-inner-branch PMEG with novel features.

## Case report

An 80-year-old man with worsening dyspnea was transferred for a 10-cm aortic arch aneurysm associated with a chronic type B aortic dissection (Society for Vascular Surgery/Society of Thoracic Surgeons type B3, 10; zones 5-10 nonaneurysmal; visceral branches spared). Imaging also revealed a 5-cm nondissected ascending aortic aneurysm and extensive arch calcification (Figs 1 and 2). His transthoracic echocardiogram demonstrated reduced left ventricular ejection fraction of 35% to 40% and reduced right ventricular systolic function. Cervical arch debranching followed by zone 0 single-branch thoracic endovascular repair was initially planned, but the procedure was aborted because of a near-arrest arrythmia during anesthetic induction. Given his advanced age, significant cardiac comorbidities, acute pulmonary symptoms, and high risk of recurrent arrhythmia during cervical debranching, a single-admission, two-stage hybrid approach was adopted. Written informed consent was obtained, including agreement for off-label use and permission to publish.

**Fig 1:**
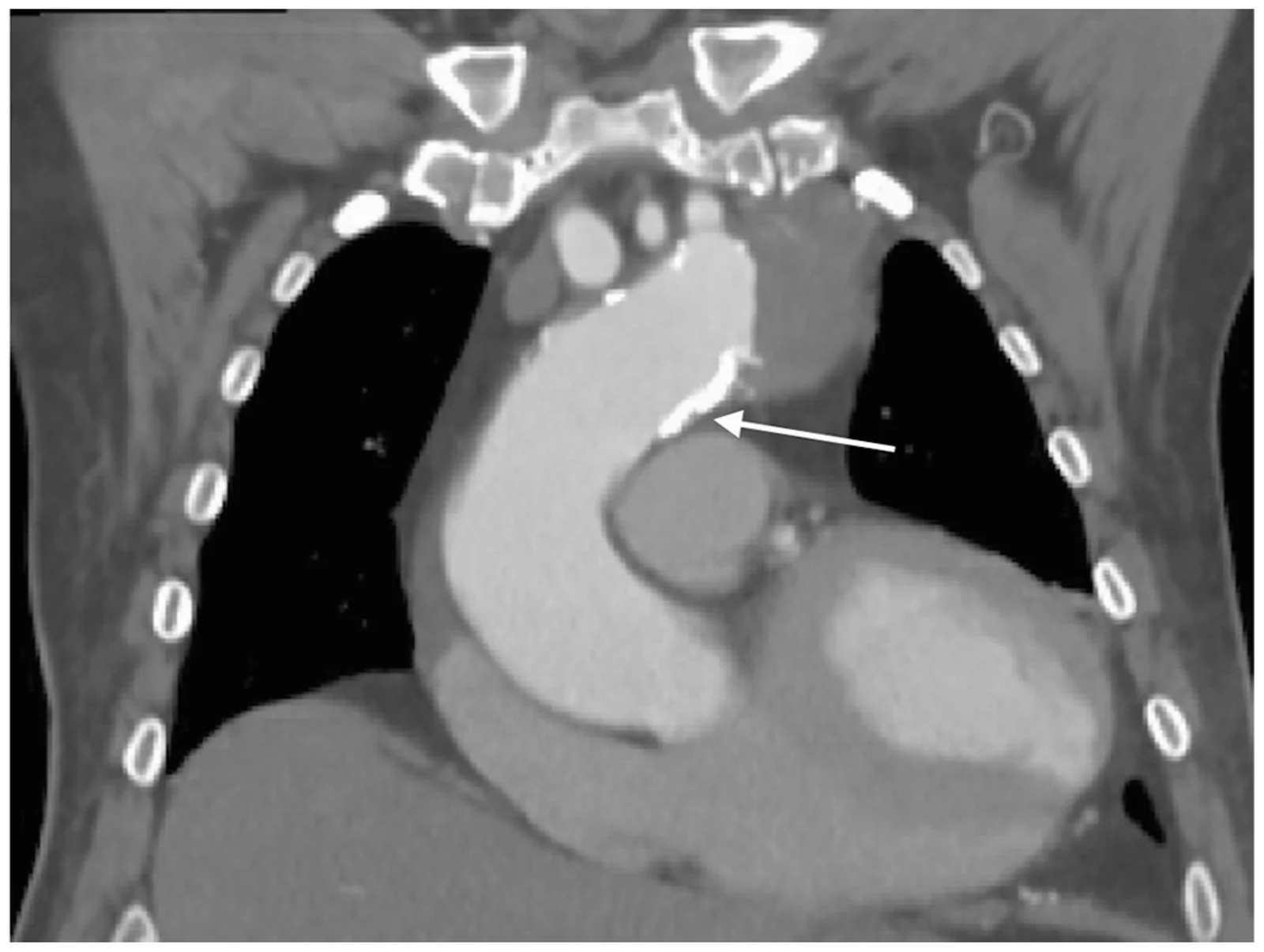
Preoperative computed tomography scan with a calcified aortic arch (*white arrow*).

**Fig 2:**
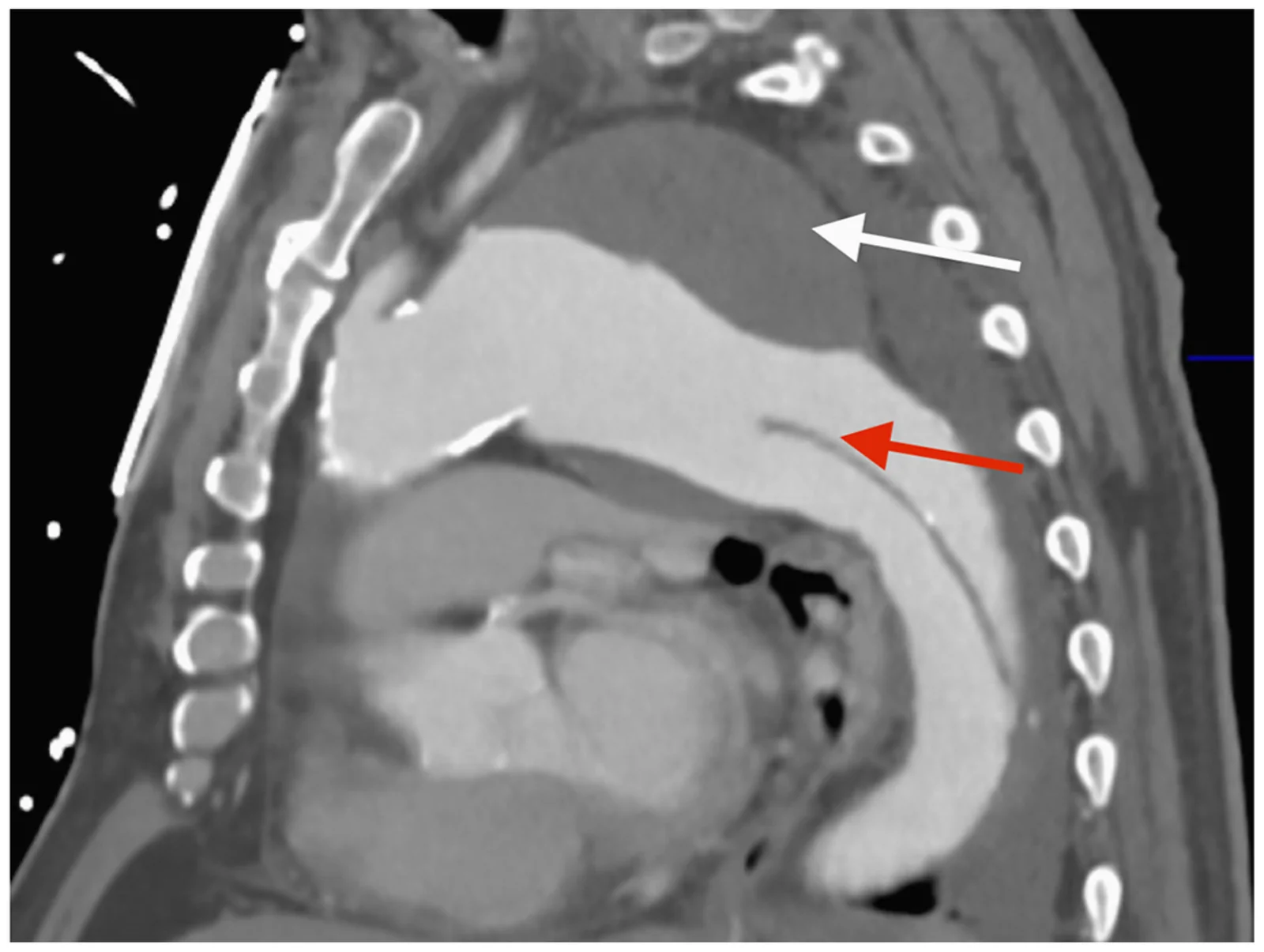
Preoperative computed tomography scan demonstrating a large distal arch aneurysm (*white arrow*) and chronic dissection (*red arrow*).

### Stage 1: Open ascending aortic replacement without circulatory arrest

#### Surgical technique

The aims of stage 1 were to (1) replace the aneurysmal ascending aorta and establish a suitable proximal landing zone, while also (2) minimizing any manipulation of the heavily calcified arch to minimize stroke risk and myocardial ischemic time. The right axillary artery was used for arterial inflow while on cardiopulmonary bypass (CPB), and epiaortic ultrasound imaging was used to confirm that our planned aortic cross-clamp site was as distal as possible without involving the calcified proximal arch. With the heart arrested, the entire ascending aorta was excised. Aortic valve competency was confirmed after resuspension, and the ascending aorta was replaced with a 30-mm Gelweave straight graft (Vascutek Ltd., Terumo Aortic, Inchinnan). To prevent kinking, final graft trimming was deferred until the heart was filled from the CPB reservoir. This ensured that the graft was measured at its correct functional length. Myocardial ischemia time was brief at 43 minutes, and the patient was separated from CPB with preserved function and no aortic insufficiency on transesophageal echocardiography. The sternum was closed with the FiberTape closure system (Arthrex) to provide sternal stability and ensure radiolucent closure, minimizing fluoroscopic artifact during the planned second-stage endovascular arch repair. The patient woke neurologically intact, extubated the day of surgery, and recovered uneventfully.

### Stage 2: Total endovascular arch repair using a three-inner-branch PMEG

#### Device modification

Using computed tomography angiography-based multiplanar reconstructions, target vessel positions were mapped via longitudinal and radial distances from a reference point at the graft’s 12 o'clock top edge. On the sterile back table, the Zenith TX2 Dissection Endovascular Graft (Cook Medical) was modified to include three inner branches, with antegrade and retrograde orientations defined relative to the main endograft and their originating fenestrations: an 11-mm fenestration for the antegrade innominate artery (IA) and 8-mm fenestrations for the antegrade left common carotid artery (LCCA) and the retrograde left subclavian artery (LSA) (Fig 3). Inner branches were constructed using 2.5-cm beveled Viabahn self-expanding covered stents with unbeveled proximal segments (W. L. Gore & Associates), outlined with radiopaque markers and reinforced with sutures (Fig 4). The two proximal portals (IA and LCCA) were positioned within the double-tapered portion of the device, where a permanent diameter-reducing tie was then placed distal to them (Fig 3).

**Fig 3:**
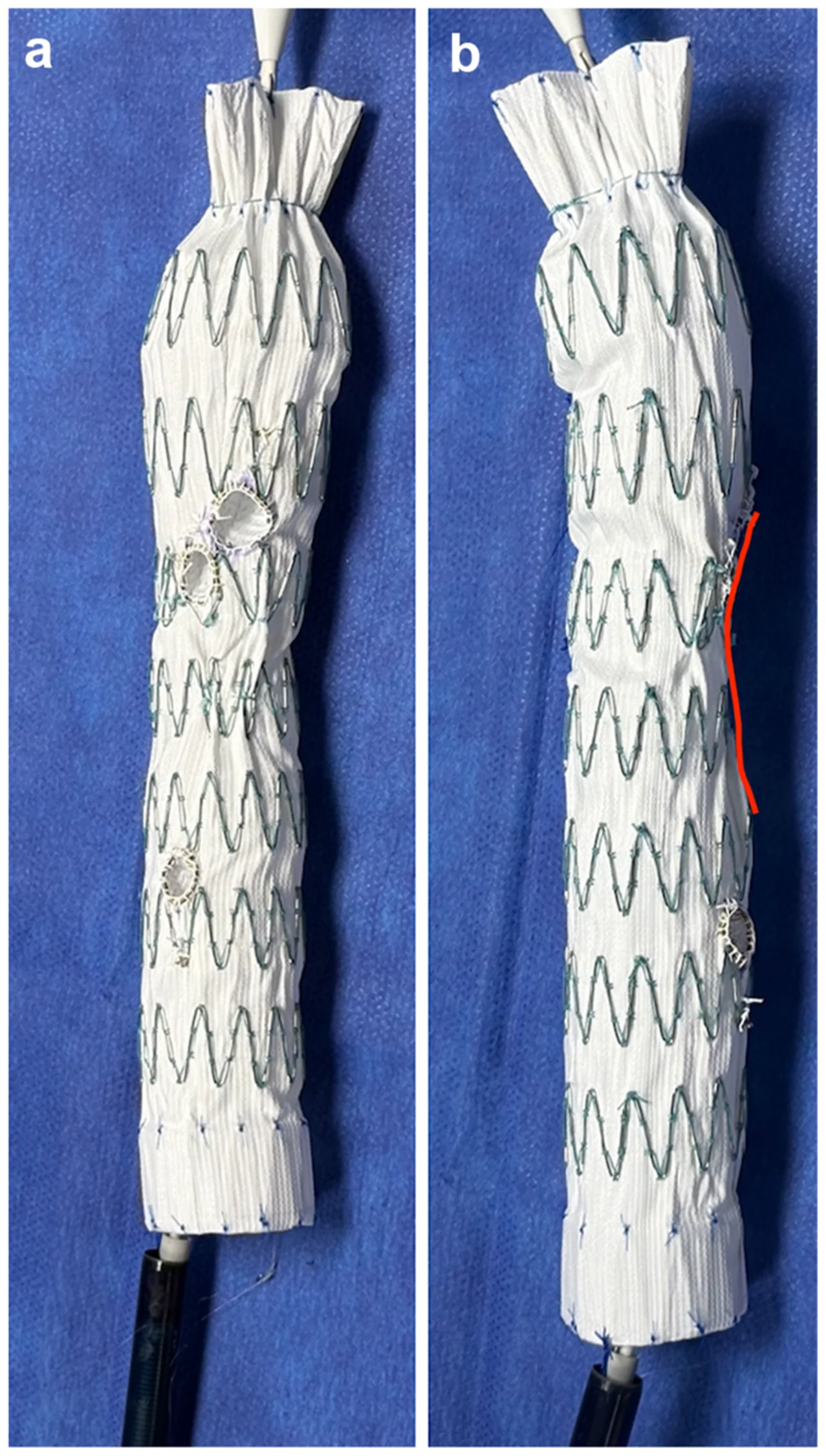
Benchtop-modified stent graft. The unsheathed device displays three fenestrations with radiopaque markers to accommodate the innominate, left common carotid, and left subclavian arteries. **(a)** Anterior view and **(b)** lateral view showing the permanent diameter-tie distal to the two proximal inner branch portals that are positioned within the double-tapered part of the device (*red line*).

**Fig 4:**
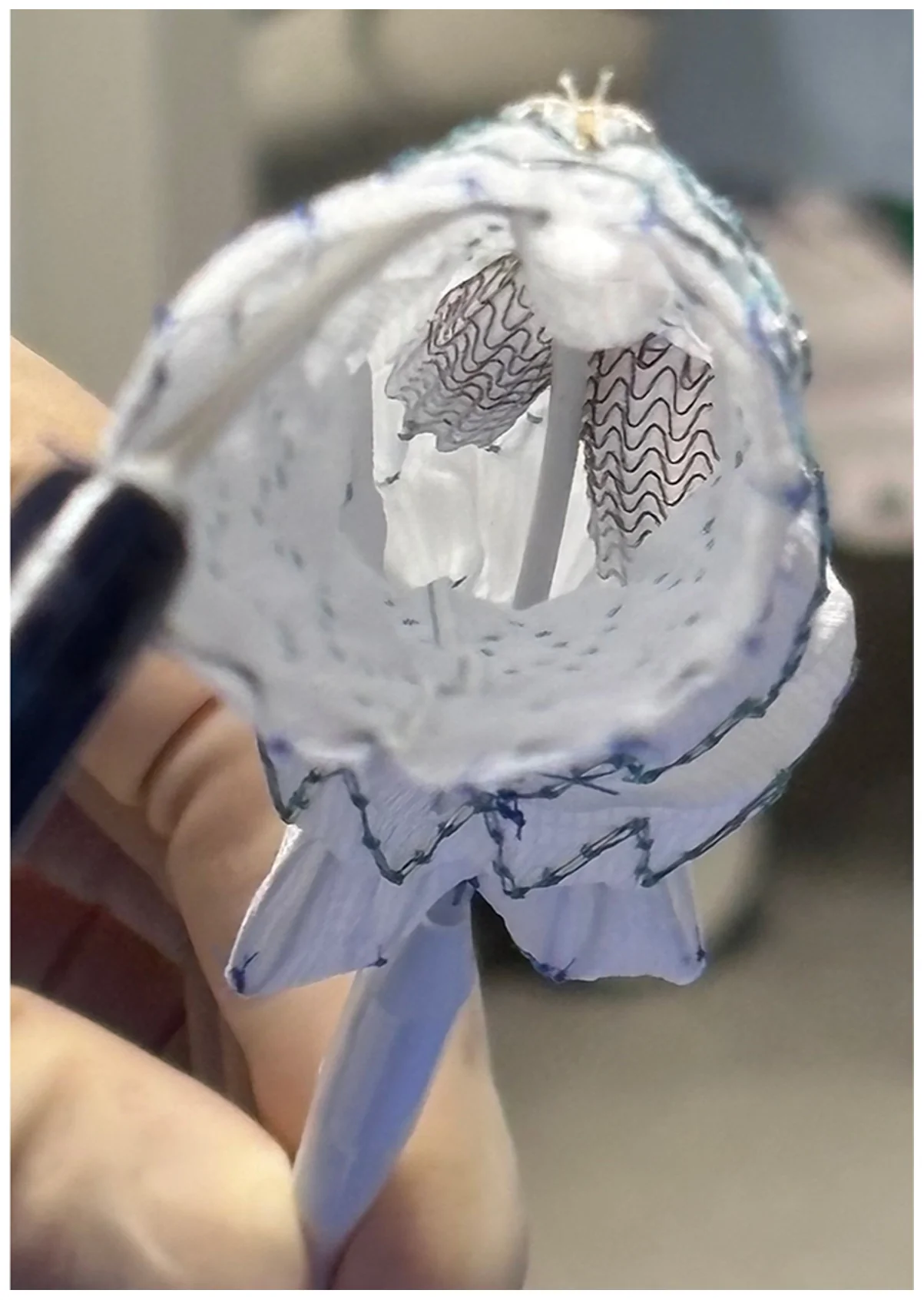
Magnified view demonstrating the three inner branches constructed using Viabahn endoprostheses and securely sutured to the main endograft fabric.

#### Implantation technique

Under ultrasound guidance, the right common femoral artery (RCFA) and left common femoral vein were accessed percutaneously. An 8F sheath was placed in the RCFA. After systemic heparinization (100 U/kg intravenous bolus; target activated clotting time, 250-300 seconds), a 12F DrySeal sheath (W.L. Gore & Associates, Inc.) was inserted into the left common femoral vein, and a Coda balloon (Cook Medical) was fluoroscopically positioned at the atriocaval junction for inflow occlusion and permissive hypotension during device deployment.

Under direct vision, bilateral carotid access was established. After exchanging the RCFA access sheath for a 22F sheath, a pigtail catheter was advanced into the left ventricle and exchanged for a Lunderquist (Cook Medical) extended double-curved wire, and the sheath was flushed to ensure that the entire system was free of air bubbles. A Zenith TX2 Dissection Endovascular Graft (Pro-Form; Cook Medical) was deployed in zone 3 and distally extended using a Zenith Dissection Endovascular Stent (Cook Medical).

After flushing with heparinized saline, the modified device was positioned in the ascending aorta and deployed during temporary atriocaval occlusion and bilateral carotid clamping for cerebral embolic protection. After clamp release, target arteries were sequentially catheterized and stented: the IA branch with a Gore thoracic branch endoprosthesis side branch (TBE-SB) (W. L. Gore & Associates) extended using a Gore Excluder Contralateral Leg, and the LCCA and LSA branches with 9-mm × 79-mm VBX stents (W. L. Gore & Associates). Completion angiograms confirmed the patency of the endograft and all branches (Fig 5). The patient recovered uneventfully, was discharged on dual antiplatelet and high-intensity statin therapy after a total 14-day hospital stay (including 1-week interval between the first- and second-stage procedure), and showed a fully patent endograft without evidence of endoleak on 1-month follow-up computed tomography angiography.

**Fig 5:**
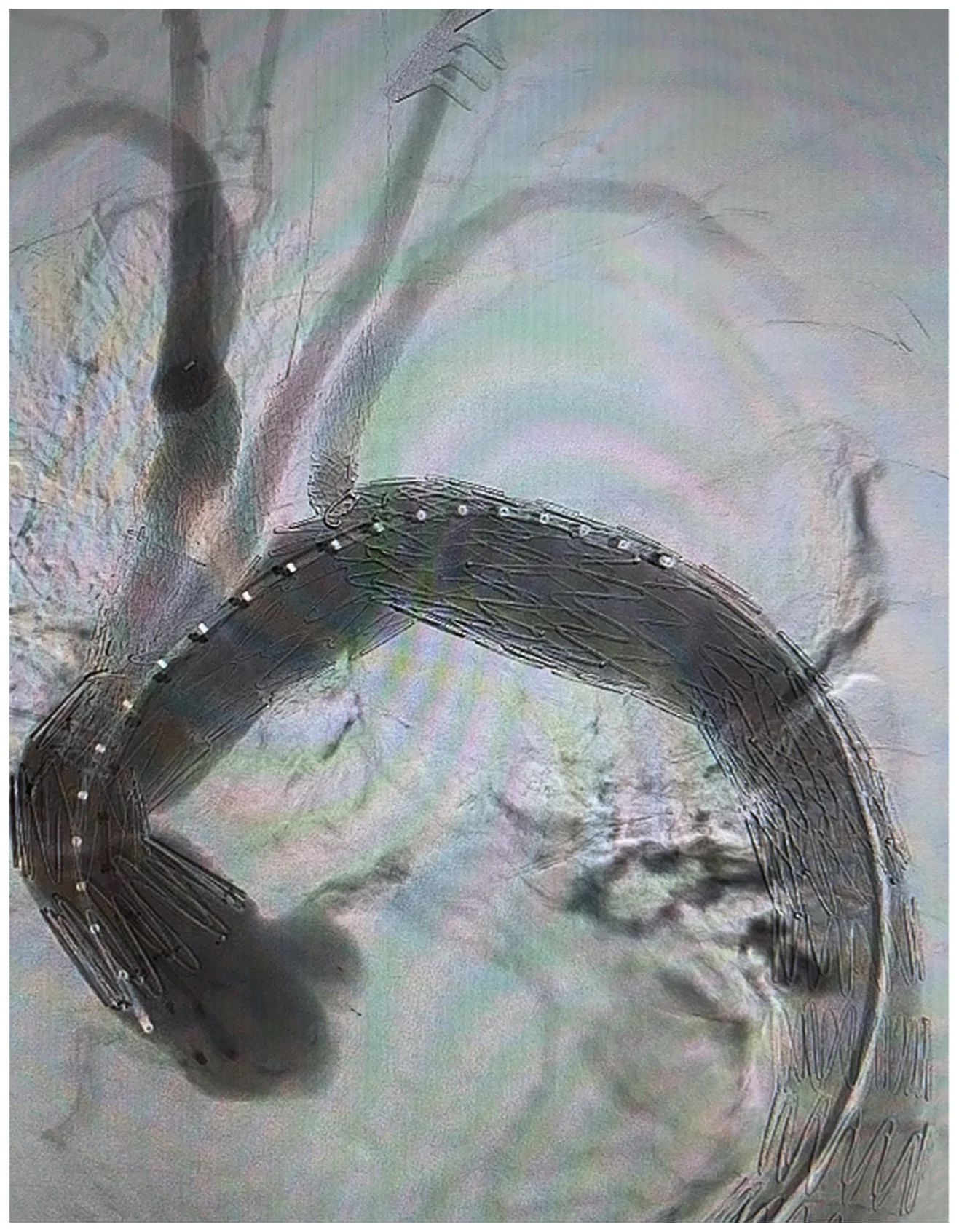
Completion angiogram.

## Discussion

While building on established techniques, our approach introduces a PMEG with novel features and technical updates that improve both safety and accessibility. Specifically, we incorporated a permanent constraining mechanism distal to the two proximal inner branch portals positioned within the double-tapered part of the device. This configuration increased the space between the endograft and the greater curvature of the aortic arch (Fig 3, *b*), enhancing procedural control and facilitating subsequent branch cannulation. Furthermore, this design maintains continuous cerebral perfusion regardless of portal alignment precision.

Key supportive techniques ensured procedural success. Given the patient’s recent severe arrhythmia and significant cardiac dysfunction, atriocaval occlusion was selected over rapid ventricular pacing to minimize cardiac motion and prevent inadvertent endograft migration.^4^ Because superior vena cava drainage preserves myocardial perfusion,^5^ this technique was well tolerated by our patient despite underlying cardiac dysfunction.

Another critical technology used was the TBE-SB. In branched endovascular aortic arch repair, the reverse-tapering morphology of the IA presents a challenging landing zone,^6^ further complicated by a lack of covered stents wider than 13 mm needed for a reliable seal. Although originally designed for LSA perfusion during zone 2 thoracic endovascular repair, the TBE-SB offers distinct advantages for IA incorporation due to its tapered profile.^7^

Notably, the Endo-Bentall procedure has emerged as a novel strategy for the total endovascular management of complex ascending aortic pathologies.^8^ Previous studies report favorable outcomes using a combined approach—an Endo-Bentall procedure followed by total endovascular arch repair—for extensive aortic dissection involving the ascending, arch, and descending aorta.^9^ However, this fully endovascular strategy was not indicated in the present case given the absence of aortic root pathology and the elevated risk of recurrent arrhythmia.

## Conclusions

This case highlights the shift toward integrated, anatomy-driven strategies for complex aortic ascending-aortic arch repair. By incorporating a permanent constraining mechanism feature on the PMEG, this hybrid strategy improves anatomical control and procedural safety.

## Funding

None.

## Disclosures

W.J.Y. is a consultant for W. L. Gore & Associates and Cook Medical. A.P.N. reports no conflicts.
